# Effects of Invasive *Solidago canadensis* and Biochar on the Remediation of Soil Cd Contamination and Greenhouse Gas Emissions

**DOI:** 10.3390/life15121927

**Published:** 2025-12-16

**Authors:** Xiaokang Ni, Yadi Yu, Xi Liu, Wanqing Nie, Yuli Hu, Jian Bai, Ziyi Yan, Wei Li, Lifei Xiong, Xixian Xie, Yuanyuan Zhu, Zihan Zeng, Qingye Yu, Shuli Wang, Qin Ying, Nansheng Wu, Ling Zhang

**Affiliations:** 1 Jiangxi Provincial Key Laboratory of Subtropical Forest Resources Cultivation, College of Forestry, Jiangxi Agricultural University, Nanchang 330045, China; 2 National Innovation Alliance of *Choerospondias axillaris*, Nanchang 330045, China

**Keywords:** biochar, cadmium contamination, greenhouse gas emissions, remediation measures, *Solidago canadensis*

## Abstract

Cadmium (Cd) contamination in agricultural soils threatens food security and exacerbates climate change through its impact on greenhouse gas (GHG) (CO_2_, N_2_O and CH_4_) emissions, in which N_2_O and CO_2_ are the dominant fluxes of the terrestrial carbon-nitrogen cycle whose magnitude is directly amplified by Cd stress. Key remediation approaches for this dual challenge are phytoremediation and biochar amendment. This study aims to investigate the effects of *Solidago canadensis* (CGR) and biochar (BC) on soil remediation and GHG emissions under different levels of Cd contamination. A pot experiment with four Cd concentration gradients (0, 5, 10, and 30 mg kg^−1^, i.e., Cd-0, Cd-5, Cd-10, and Cd-30, respectively) and three remediation measures (control, BC addition, and CGR cultivation) was set up to measure available soil Cd (ACd), soil physicochemical properties, GHG emissions, and plant Cd accumulations. The results demonstrated that ACd was significantly reduced by BC via adsorption through surface complexation and by CGR via immobilization through root uptake and sequestration. CGR decreased ACd by 46.2% and 41.7% under mild and moderate Cd contamination, respectively, while BC reduced ACd by 8.9% under severe contamination. In terms of GHG emissions, CGR increased cumulative CO_2_ by 83.4% in Cd-10 soil and 53.8% in Cd-30 soil, whereas BC significantly lowered N_2_O emissions by 22.1% in Cd-5 soil. Mantel analysis revealed strong correlations between ACd and key carbon and nitrogen indicators, which mediate the bioavailability of Cd. Therefore, CGR cultivation is better suited to mild-to-moderate contamination given its high removal efficiency, while BC amendment is targeted at severe contamination by stabilizing Cd and mitigating N_2_O. This provides a scientific basis for the remediation of Cd-contaminated soils.

## 1. Introduction

Cadmium (Cd) is a highly toxic metallic element characterized by high mobility, long persistence, and wide-ranging environmental impacts. It is listed as one of the globally prioritized pollutants for control [1]. Its primary sources include natural processes and human activities, such as applying cadmium-containing pesticides and fertilizers in agriculture [2]. In China, the situation of Cd pollution is particularly severe. According to the 2014 National Soil Pollution Status Survey Bulletin, the exceedance rate of Cd in arable soils reached approximately 7%, the highest among all heavy metal pollutants [3]. Notably, southern Chinese provinces form the core of Cd pollution, with areas like Guangxi, Hunan, Fujian, and Jiangxi exceeding safe cadmium limits by 3 to 4 times [4]. Therefore, the remediation of Cd-contaminated soil is now a major focus of national and international research. Strategies widely under investigation include soil remediation technologies (e.g., phytoremediation) and amendments (e.g., biochar), which aim to lower Cd bioavailability and secure agricultural safety [5].

Soil Cd contamination not only poses a potential threat to human health but also contributes to increased greenhouse gas (GHG) emissions, thereby affecting agricultural production and ecosystem stability [6]. In global GHG emissions, carbon dioxide (CO_2_), methane (CH_4_), and nitrous oxide (N_2_O) are widely recognized as the major GHGs that cause global warming [7]. Agricultural soils account for approximately 60% of anthropogenic N_2_O emissions [8]. Therefore, soil processes play a crucial role in the overall greenhouse effect. The long-term Emission Database for Global Atmospheric Research (EDGAR) further shows that global emissions of CO_2_, CH_4_, and N_2_O have continued to rise from the early 1970s to 2022. Over the 40-year period from 1980 to 2020, total anthropogenic N_2_O emissions increased by 40%, indicating that N_2_O is an important GHG in addition to CO_2_ [9]. Meanwhile, the annual average growth rate of global atmospheric CO_2_ amounts has reached 2.2% since 1960 [10]. Although existing research indicates that Cd contamination can indirectly increase GHG emissions by stressing soil microbes and disrupting carbon and nitrogen cycles, the precise regulatory mechanisms remain unclear [11,12].

Traditional soil Cd remediation methods are often costly and have limited efficacy. Therefore, developing economical and effective soil Cd remediation technologies is particularly critical [13]. Biochar is a stable, black, carbonaceous material produced through the pyrolysis of biomass at 300–700 °C in an oxygen-deficient environment [14]. Relevant studies have shown that biochar has a certain adsorption capacity for heavy metals (Cd, Hg, Pb, Cu, Ni, Zn, As, Cr) [15]. Biochar benefits from abundant raw material sources and low production costs [16]. Moreover, its high specific surface area, porous structure, and abundance of surface functional groups enable the effective reduction in soil Cd bioavailability and mobility via adsorption and complexation [17]. Relevant data demonstrate that biochar application significantly decreases soil-available Cd and plant Cd accumulation, with average reductions of 52% and 38%, respectively [18]. Functional groups on the biochar surface can form stable complexes with Cd, which further diminishes Cd mobility and bioavailability [19]. Researchers found that a 3% (*w*/*w*) application of rapeseed straw biochar significantly altered soil Cd speciation, thereby reducing its mobility [20]. Other studies have also indicated that biochar can improve soil pH, pore structure, and microbial activity, which in turn enhances soil quality and increases crop yields [21]. In addition, biochar can mitigate GHG emissions and strengthen soil carbon-nitrogen retention by regulating soil enzyme activity and the abundance of microbial functional genes [22,23]. Thus, biochar is a soil amendment and Cd-contamination remediation material with great application prospects.

*Solidago canadensis* (henceforth *S. canadensis*), native to the eastern and north-central regions of North America, is a perennial herb of the genus *Solidago* (Asteraceae family) that thrives under moderate-temperature conditions. It is recognized as one of the most destructive invasive plant species [24,25,26]. Owing to its rapid growth, broad adaptability, and strong reproductive capacity, *S. canadensis* readily forms monodominant stands, outcompeting native plants, altering vegetation structure, and ultimately threatening biodiversity and ecosystem stability [27,28]. As an invasive plant with strong environmental adaptability and vigorous growth, *S. canadensis* exhibits strong adsorption and bioaccumulation capacities for heavy metals in the soil. Research has demonstrated that *S. canadensis* is capable of adsorbing significant quantities of copper (Cu) from soil, which highlights its high potential as a species for phytoremediation of soils polluted by heavy metals [29]. Biochar, which possesses functions of carbon sequestration, emission mitigation, and heavy metal adsorption, is widely used as a soil amendment [30,31].However, whether interactions between the growth characteristics of *S. canadensis* and biochar can be utilized for the remediation of Cd-contaminated soil remains a scientific question that requires clarification. Against this background, this study proposed the following questions: (1) How does the remediation efficacy of *S. canadensis* compare with that of biochar across a gradient of soil Cd pollution levels? (2) How do applications of *S. canadensis* and biochar contrast in their impacts on soil CO_2_ and N_2_O emissions under varying Cd pollution levels?

## 2. Materials and Methods

### 2.1. Description of the Study Site

The experimental site is located in the Agricultural Science and Technology Park of Jiangxi Agricultural University, Nanchang, Jiangxi, China (115°49′52.49″ E, 28°46′17.28″ N). It has a mid-subtropical warm-humid monsoon climate, with an average annual temperature ranging from 16.3 °C to 19.5 °C. The maximum summer temperature can reach 40 °C, while the minimum winter temperature can drop to -10 °C. The annual precipitation ranges from 1341 mm to 1943 mm, with an uneven distribution throughout the year; the majority of precipitation occurs from April to June.

### 2.2. Experimental Materials

#### 2.2.1. Preparation of Biochar

*Camellia oleifera* is widely cultivated in China, with large quantities of fruit shells discarded as waste after oil extraction. Converting these fruit shells into biochar not only enables efficient resource recycling but also significantly alleviates waste disposal pressure. Here, fruit shells were collected, washed to remove debris, and air-dried in a well-ventilated location. After drying, the shells were crushed using a high-speed rotary pulverizer and passed through a 2 mm sieve to ensure uniform particle size. Subsequently, the sieved shell powder was placed into a crucible, which was then transferred to a muffle furnace for pyrolysis at 600 °C for 1 h under oxygen-limited conditions. After pyrolysis, the resulting biochar (pH: 9.49 ± 0.33, TC: 752.87 ± 3.50 g kg^−1^,TN: 5.13 ± 0.81 g kg^−1^) was allowed to cool naturally to room temperature, re-sieved through a 2 mm sieve, and finally stored in a sealed container for subsequent experimental use [32].

#### 2.2.2. Soil Sample Preparation

Soils used in this experiment were collected from farmland in the Science and Technology Park of Jiangxi Agricultural University. A random multi-point sampling method was adopted, with a soil sampling depth of 0–20 cm to ensure the representativeness of soil samples. After collection, stones, plant and animal residues, and plant roots were removed, followed by passing the soil through a 2 mm sieve and thorough mixing. To establish soil environments with different Cd contamination levels, 1.5 kg of homogenized soil was used per sample. Specifically, 0 mL, 7.5 mL, 15 mL, and 45 mL volumes of the 1000 mg L^−1^ CdCl_2_ stock solution were added to the soil samples, respectively. Deionized water was then supplemented to bring the total liquid volume to 45 mL, resulting in soil samples with four target Cd concentrations (0, 5, 10, and 30 mg kg^−1^) [33]. These Cd-spiked soils were thoroughly mixed, then sealed and incubated in the dark for 70 days to allow Cd ions to be evenly distributed and to better simulate actual field soil conditions. The stabilized soils were subsequently used for pot experiments. Soil background values are presented in Table 1.

#### 2.2.3. Selection of Test Plants

Seedlings of the test plant *S. canadensis* were collected from wild populations in Jiangxi Province. To ensure consistency, we selected healthy, current-year seedlings with a uniform height of approximately 18 cm for use in the experiments. After collection, the seedlings were promptly transported to the experimental site, where the soil adhering to the roots was removed and the seedlings were rinsed thoroughly. They were then placed in a cool and well-ventilated area for later use.

### 2.3. Experimental Design

A pot experiment was conducted using a full factorial experimental design with two factors: (i) Cd concentration and (ii) soil remediation measures. The experiment was conducted under natural, well-ventilated conditions. Plants were watered regularly to maintain soil moisture at 60–70% of field capacity. Cd concentrations were set at 0 mg kg^−1^ (Cd-0, None), 5 mg kg^−1^ (Cd-5, Mild), 10 mg kg^−1^ (Cd-10, Moderate), and 30 mg kg^−1^ (Cd-30, Severe). The three soil remediation measures including control (CK), fruit shell biochar (BC), and *S. canadensis* planting (CGR). Each remediation measures combination had 8 replications, resulting in a total of 4 × 3 × 8 = 96 pots, among which, 4 replicates per combination were allocated to gas sampling, and the remaining 4 to soil sampling. Uniform black plastic pots were used, with a 16 cm top diameter, 11 cm bottom diameter, and 18.1 cm height. Each pot was filled with 1.5 kg of pre-treated, Cd-spiked soil (stabilized prior to use) corresponding to the assigned Cd concentration. For the BC remediation measure, *Camellia* shell biochar was mixed into the Cd-spiked soil at a rate of 20 g kg^−1^ soil before potting. The CGR remediation measure involved planting a single *S. canadensis* seedling in each pot after soil preparation. In contrast, the CK pots contained soil only, with no plant or biochar amendments. The experiment began after the transplanted *S. canadensis* plants had established, as indicated by new leaf growth in at least 90% of individuals, and continued for a 150-day period.

#### 2.3.1. Gas Sampling and Analysis

Soil N_2_O and CO_2_ emissions were measured using an opaque static chamber (21.1 cm diameter × 66.9 cm height), which featured an open-bottomed cylindrical design. The outer surface of the cylindrical chamber was wrapped with metal foil to minimize the impact of solar radiation during gas sampling. Two holes were made in the chamber: one at the top, and another 23 cm above the chamber bottom on the side. The top hole was fitted with a thermometer (inserted through a rubber stopper) for temperature measurement, while the side hole was connected to an internal hose (leading to the chamber top) and an external three-way valve. Gas samples were collected once every 7 days under clear weather conditions. To ensure airtightness during sampling, an appropriate volume of water was added to a tray, a base was placed inside the tray, and the potted plant was positioned on the base; the static chamber was then placed over the pot, Throughout the entire gas-sampling period, the chamber remained undisturbed to maintain an airtight seal. At 0, 10, 20, and 30 min after chamber placement, a 60 mL plastic syringe was used to extract gas, and the corresponding chamber temperature was recorded at each time point. Pressure and temperature fluctuations inside the chamber were corrected in the calculation. Four gas samples were collected per pot, sealed in the syringe, and promptly transported to the laboratory for concentration analysis via gas chromatography (Agilent 7890B, Santa Clara, CA, USA) equipped with a flame ionization detector (FID) and an electron capture detector (ECD). During gas sampling, atmospheric temperature, soil temperature (at 10 cm depth), and soil moisture (measured using a Hydro-Sense sensor, Campbell Scientific, Logan, UT, USA) were also recorded. The calculation equations for soil N_2_O and CO_2_ emission fluxes are as follows [34]:

(1) $$F\;=\;P\times V\cdot \frac{dc}{dt}\times \frac{1}{RT}\times \frac{1}{A}\times M$$

In the formula, *F* represents the emission fluxes of N_2_O and CO_2_ (μg m^−2^ h^−1^); *P* denotes the atmospheric pressure under standard conditions (pa); *V* and *A* are the volume (m^3^) and the base area (m^2^) of the static chamber, respectively; *R* is the universal gas constant, with a fixed 8.314 J·mol^−1^·k^−1^; *dc/dt* is the rate of change in gas concentration inside the chamber with time per unit time. over time; *T* represents the absolute temperature during the sampling process (K); *M* is the relative molecular mass. The calculation formulas for the cumulative emissions of soil N_2_O and CO_2_ are as follows [35]:

(2) $$M\;=\;\sum (F_{i+1}+F_{i})/2\times (t_{i+1}-t_{i})\times 24$$

In the formula, *M* represents the cumulative gas emissions (CO_2_ cumulative emissions are measured in mg m^−2^, N_2_O cumulative emissions in μg m^−2^); *F* represents the gas emission flux (CO_2_ emission flux is measured in mg m^−2^ h^−1^, N_2_O gas e mission flux is in μg m^−2^ h^−1^); (*F_i+_*_1_ + *F_i_*) represents the sum of the gas emission fluxes from two consecutive samplings at the same sampling point, where *i* represents the *ith* gas sample collection, and (*t_i+_*_1_ *− t_i_*) represents the number of days between two consecutive samplings.

#### 2.3.2. Soil Sampling and Analysis

Soil samples were collected once a month for a total of five months. For each treatment, three soil cores (using a 1.5 cm-diameter small soil auger) were collected from three random points in each of the four replicate pots; these cores were then mixed into a composite sample, resulting in a total of 48 samples. Plant roots and gravel impurities were removed from the soil samples, which were subsequently passed through a 2 mm sieve, a portion of the samples was stored at 4 °C for refrigeration, while the remainder was air-dried at ambient temperature, and preserved for no more than 7 days prior to chemical analysis. Before the analysis, for analyses conducted on fresh soil, the moisture content was first determined and used to convert all results to a dry-soil basis; subsequently, each assay was performed using an equivalent dry-mass of fresh soil to ensure consistency. Soil pH was measure by a pH meter (PH-3E). Total carbon (TC), dissolved organic carbon (DOC), and dissolved organic nitrogen (DON) in the soil were determined using a total organic carbon analyzer (Analytik Jena, Multi N/C 3100, Jena, Germany) [36]. For soil total nitrogen (TN) and ammonium nitrogen (NH_4_^+^), an automatic discrete chemical analyzer (Smart Chem 200, Westco, Rome, Italy) was employed [37]. Nitrate nitrogen (NO_3_^−^) was tested via a flame photometer. Available cadmium (ACd) was extracted following the BCR method (European Community Bureau of Reference), and its concentration was analyzed by inductively coupled plasma mass spectrometry (ICP-MS); soil samples were tested both at the start and end of the experiment. After the experiment, the ACd concentrations in the roots, stems, and leaves of plant samples were determined, and their absorption capacity was calculated using the following formula:

(3) Q = C × B

In the formula, *Q* represents the Cd absorption of plant (mg); *C* represents the cadmium concentration in plants (mg kg), and *B* represents the plant biomass (mg).

#### 2.3.3. Statistical Analysis

Two-way analysis of variance (ANOVA) was used to examine the effects of remediation measures, Cd concentration, and their interaction on each index. Normality and homogeneity were conducted on all measured indices to ensure that the statistical analysis prerequisites were met. For significant differences, post hoc multiple comparisons (Duncan method) were conducted to compare specific differences among means. Correlations among ACd and soil physicochemical properties were elucidated by Mantel analysis. All statistical analyses were completed using SPSS 27.0 and R.4.4.3 software.

## 3. Results

### 3.1. Soil Physicochemical Properties

**Figure 1:**
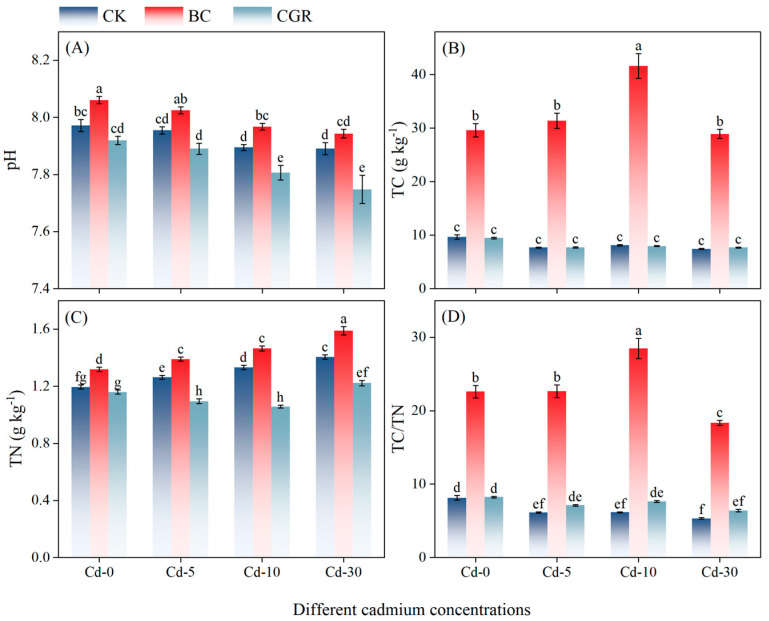
The effects of remediation of Cd-contaminated soil under different remediation measures on soil pH (**A**), TC (**B**), TN (**C**), TC/TN ratio (**D**). Different letters indicate significant differences. CK: control; BC: biochar; CGR: *S. canadensis* cultivation. Cd-0: 0 mg kg^−1^; Cd-5: 5 mg kg^−1^; Cd-10: 0 mg kg^−1^; Cd-30: 30 mg kg^−1^, the same below.

Both remediation measures and Cd concentrations exerted extremely significant effects on TC and TN (*p* < 0.001; Table 2). Under different Cd concentrations, the application of BC significantly increased the content of TC (Figure 1B), with an increase of 207.45% to 412.3% compared to the CK, and the TN content of the BC was also higher than that of the CK and CGR (Figure 1C).

**Figure 2:**
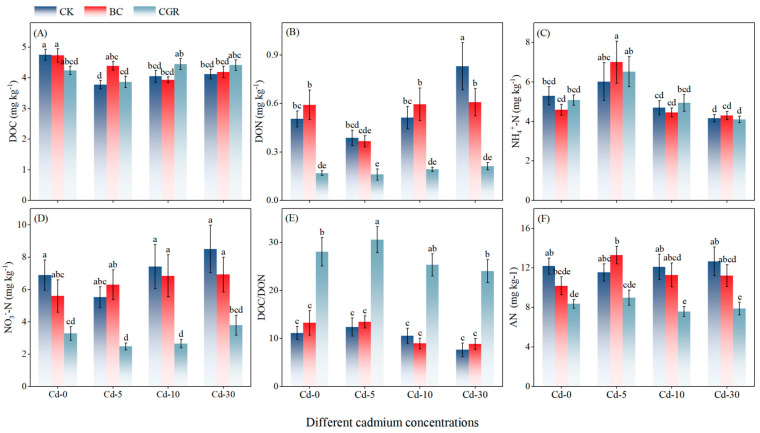
The effects of remediation of Cd-contaminated soil under different remediation measures on soil DOC (**A**), DON (**B**), NH_4_^+^-N (**C**), NO_3_^−^-N (**D**), DOC/DON ratio (**E**), and AN (**F**). Different letters indicate significant differences.

DOC exhibited a declining trend with increasing Cd concentrations (Figure 2A). At Cd-10 and Cd-30 levels, DOC content was significantly higher in the CGR than in the BC. For DON (Figure 2B), concentrations in the CGR were lower than in the CK across all Cd levels. In the BC treatment, DON was 17.3% higher than in CK at Cd-0, but comparable to CK at Cd-30. Regarding inorganic nitrogen, NO_3_^−^-N content in the BC treatment exceeded that in CGR by 154.7% at Cd-5 and 159.1% at Cd-10 (Figure 2D). Meanwhile, NH_4_^+^-N in BC was 16.3% higher than in CK at Cd-5 (Figure 2C). For available nitrogen (AN; Figure 2F), no significant difference was observed between CGR and CK across Cd concentrations, while AN content in BC was higher than in CGR. Finally, the CGR treatment elevated the DOC/DON ratio compared to both BC and CK at all Cd levels (Figure 2E). This ratio peaked at Cd-5, where it was 147.3% higher than in CK.

### 3.2. Soil-Available Cadmium

**Figure 3:**
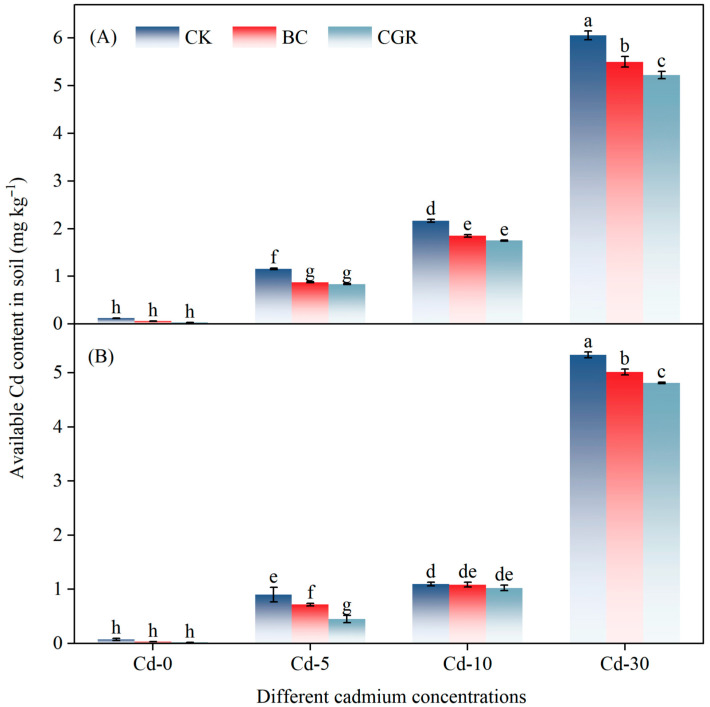
The effects of remediation of Cd-contaminated soil under different remediation measures on soil-available cadmium (at the initial stage of the experiment, (**A**)) and (at the end of the experiment, (**B**)). Different letters indicate significant differences.

**Figure 4:**
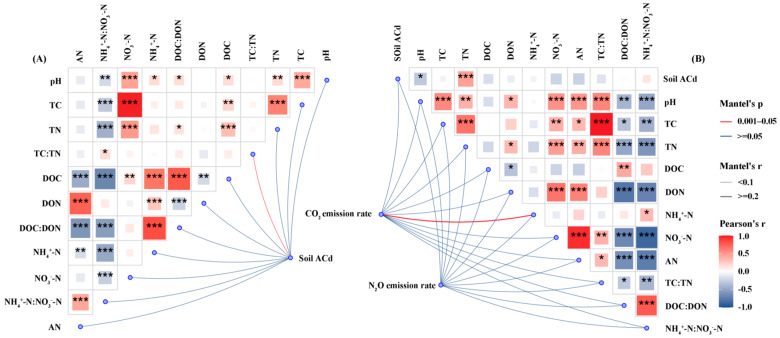
Mantel test and Pearson correlations between soil-available Cd (**A**), GHG emission rates (N_2_O and CO_2_) (**B**), and measured soil variables across all remediation measures and Cd contamination levels. The soil variables include basic physicochemical properties, carbon and nitrogen components, and inorganic nitrogen forms. Line width and color in the network diagram correspond to the strength and significance of the correlations, respectively. *, *p* < 0.05; **, *p* < 0.01, ***, *p* < 0.001.

Both remediation measures and cadmium concentrations had a highly significant effect on ACd in the soil (*p* < 0.001; Table 2). Under Cd-5 and Cd-10, the ACd content in soils treated with BC or CGR was lower than that in the CK Under Cd-30, at the experiment initiation (Figure 3A), BC reduced soil ACd by 9.8% relative to CK, while CGR reduced it by 13.8%; at the experiment termination (Figure 3B), BC and CGR decreased ACd by 5.9% and 9.7%, respectively, compared with CK. ACd was significantly and positively correlated with several soil physicochemical properties (Figure 4A). Among these properties, the Mantel tests for TC and TN with ACd reached an extremely significant level (*p* < 0.001), with Pearson correlation coefficients close to 1.0. DOC and DON were also extremely significantly and positively correlated with ACd (*p* < 0.001). In addition, NH_4_^+^-N and AN also showed an extremely significant correlation with ACd (*p* < 0.001).

### 3.3. Plant Cadmium Content

**Figure 5:**
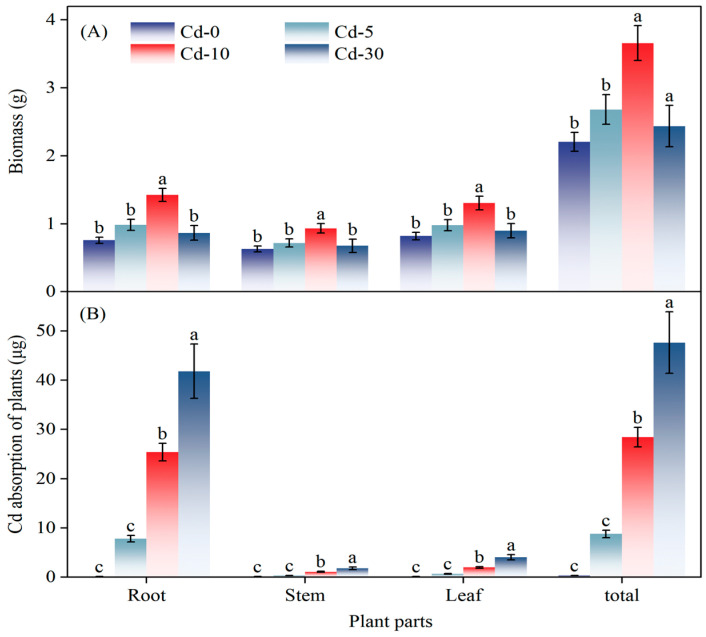
Effect of Cd-contaminated level on (**A**) biomass production and (**B**) Cd accumulation in different tissues of *S. canadensis*. Different letters indicate significant differences.

Under Cd-10 conditions, CGR produced significantly greater total and root biomass compared to other treatments, though no inter-treatment differences were observed in stem or leaf biomass (Figure 5A). Cd uptake in CGR plants increased significantly with soil Cd concentration, peaking at the Cd-30 level. Root Cd content at Cd-30 was 64.6% higher than at Cd-10. Roots served as the primary site of accumulation: per-plant root Cd uptake reached 41.8 μg under Cd-30, exceeding all other treatments. Across all Cd levels, stem and leaf Cd absorption remained lower than in the roots (Figure 5B).

### 3.4. Effect on Soil GHG

#### 3.4.1. CO_2_ Emission

**Figure 6:**
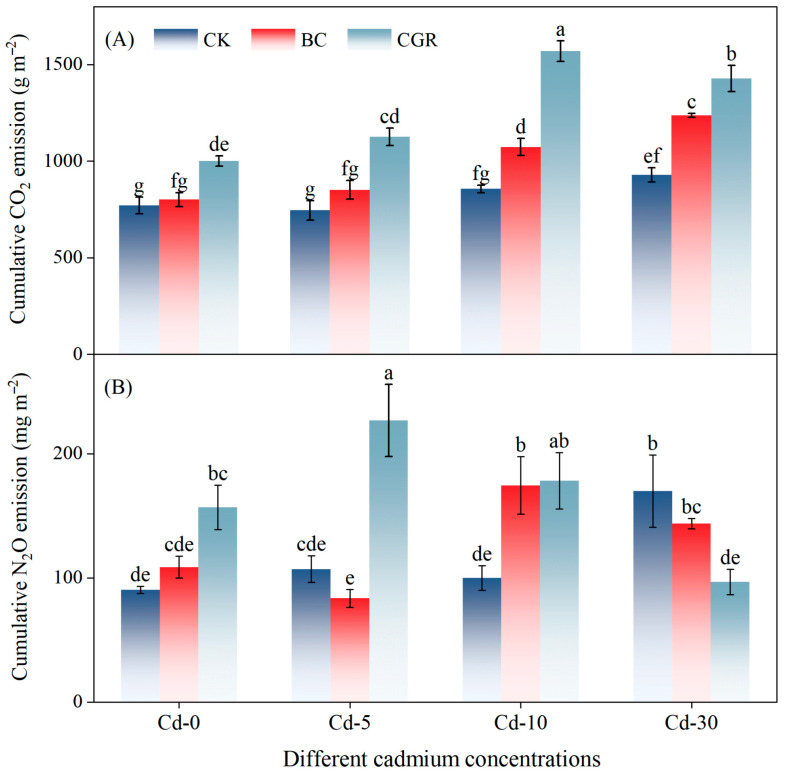
Effects of different remediation measures on cumulative emissions of soil CO_2_ (**A**) and N_2_O (**B**). Different letters indicate significant differences.

**Figure A1:**
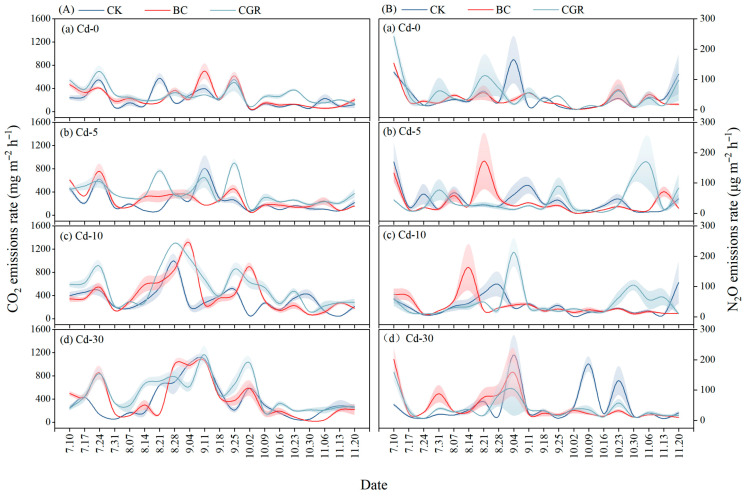
The effects of different remediation measures and cadmium concentration treatments on the dynamic changes in soil CO_2_ (**A**) and N_2_O (**B**) emission rates in cadmium-contaminated soil. The shaded area indicates the standard error of the mean.

CO_2_ emissions were significantly influenced by remediation measures, Cd concentration, and their interaction (*p* < 0.001; Table 3). The BC reduced the CO_2_ emission rate, whereas CGR significantly increased it—an effect that intensified with higher Cd contamination (Figure A1A). Under moderate and severe contamination, cumulative CO_2_ emissions in the CGR treatment were 83.4% and 53.8% higher than in the CK, respectively, and were also significantly greater than in the BC (Figure 6A).

#### 3.4.2. N_2_O Emission

Cumulative N_2_O emissions were significantly influenced by remediation measures (*p* < 0.001), Cd concentration (*p* < 0.05), and their interaction (*p* < 0.001) (Table 3). In Cd-30 soils, the BC increased the N_2_O emission rate, while the CGR inhibited N_2_O emissions in Cd-10 soils (Figure A1B). At Cd-5, cumulative N_2_O emissions in the CGR were 172.3% higher than those in the BC and 111.2% higher than those in the CK. At Cd-10, cumulative N_2_O emissions in the CGR and BC were 78.4% and 74.8% higher than those in the CK, respectively. The CGR promoted N_2_O emissions at Cd-5 but inhibited them at Cd-10 and Cd-30; in contrast, the BC generally inhibited N_2_O emissions or had no significant effect on them (Figure 6B).

The N_2_O emission rate positively correlated with soil pH, TC, TN, NO_3_^−^-N, AN, TC/TN ratio, DOC/DON ratio, and NH_4_^+^-N/NO_3_^−^-N ratio (*p* < 0.001), and positively correlated with dissolved DON and NH_4_^+^-N (*p* < 0.05). Additionally, the spatial distribution of N_2_O emission rates was significantly coupled with NO_3_^−^-N (*p* < 0.001–0.05). The CO_2_ emission rate correlated positively with NO_3_^−^-N, AN, TC/TN ratio, DOC/DON ratio, and NH_4_^+^-N/NO_3_^−^-N ratio (*p* < 0.01), with an extremely strong correlation with NO_3_^−^-N (*p* < 0.001; Figure 4B).

## 4. Discussion

### 4.1. Remediation Effects of CGR and BC on Cd-Contaminated Soil

Heavy metal Cd contamination seriously threatens soil and food security, making effective remediation materials a key research focus. CGR shows substantial remediation potential across Cd-contaminated soils, consistent with Fu et al. [38] and further validates the application value of invasive plants in heavy metal remediation [15]. Its mechanism relies on rhizosphere microenvironment regulation and enrichment, with stronger efficacy in severely polluted soils [39], confirming our initial hypothesis. Under Cd-10 and Cd-30 levels, DOC was significantly higher in CGR soils than in BC soils, likely due to root-secreted low-molecular-weight organic acids that elevate DOC and enhance Cd immobilization [40]. Meanwhile, CGR’s DON uptake enhances rhizosphere microbial activity, promoting Cd transformation and immobilization. This finding aligns with the theory of “rhizodeposition-driven rhizosphere nutrient cycling” [41].

The remediation advantage of CGR in Cd-contaminated soil stems from its “root enrichment–low above-ground translocation” trait [42]. Cellulose and pectin carboxyl groups in CGR root cell walls intercept Cd^2+^ via ion exchange, and cortical Casparian strips physically block Cd^2+^ transport to the xylem—a mechanism experimentally confirmed in multiple crops [43,44]. A small fraction of Cd^2+^ entering the cytoplasm forms stable Cd-phytochelatin (Cd-PC) complexes with PCs, which ABCC-type transporters pump into vacuoles for compartmentalization [45]. Vacuolar compartmentalization reduces Cd^2+^ toxicity and facilitates long-term immobilization. Consequently, Cd levels in CGR shoots remain minimal, preventing food chain transfer and allowing for continuous sequestration. However, the long-term field stability and growth-cycle dynamics of CGR remediation still require verification through extended field observation. This study’s validation of CGR’s remediation efficacy aligns with Richardson et al.’s view that invasive plants are potential remediation materials owing to their stress tolerance and enrichment capacity [46].

In contrast to CGR’s bioremediation mechanism, BC remediates Cd-contaminated soil through the dual effects of physicochemical passivation and soil improvement. This synergistic action lowers Cd bioavailability while enhancing soil fertility, a finding consistent with existing research [47]. This study shows BC significantly increases soil carbon and nitrogen pools: under different Cd contamination levels, its TC content is notably higher than CK, benefiting from its aromatic stable carbon skeleton and well-developed porous structure that reduce the mineralization loss of soil organic carbon, with a high specific surface area of 246.18 m^2^ g^−1^ further strengthening the carbon sequestration effect [48]. This is also confirmed by Chen et al., who reported that BC reduces organic carbon decomposition and loss by increasing soil aromatic carbon ratio and aggregate stability [30]. Meanwhile, BC electrostatically adsorbs NH_4_^+^-N via surface negative charges and may activate urease, significantly increasing TN and AN content—consistent with Ren et al.’s reported nutrient enhancement [49]. Enhanced carbon and nitrogen pools reduce nutrient leaching and improve soil physicochemical properties, creating favorable conditions for Cd passivation [50]. BC reduces soil-available Cd mainly through three mechanisms: (1) its porous structure physically intercepts Cd^2+^ to prevent soil matrix infiltration; (2) surface functional groups (e.g., -OH, -COOH) form stable complexes with Cd^2+^ via coordination bonds to strengthen adsorption; and (3) increased AN promotes NH_4_^+^ and Cd^2+^ competition for adsorption sites, further lowering Cd bioavailability [51]. However, this study’s short-term observations limit our assessment of biochar aging and long-term Cd fixation; extended field trials are needed to systematically evaluate remediation sustainability.

### 4.2. Effects of CGR and BC on CO_2_ and N_2_O Emissions

For the second hypothesis, the driving patterns of GHG emissions differed markedly between CGR and BC. In alignment with our experimental hypothesis, CGR significantly promoted soil CO_2_ emissions, with the emission levels increasing alongside higher cadmium concentrations. This positive concentration-dependent stimulatory effect further corroborates the global consensus that invasive plants generally enhance soil CO_2_ emissions [52]. This phenomenon stems from two mechanisms: first, CGR roots directly release CO_2_ via respiration and secrete low-molecular-weight organic acids to boost rhizosphere DOC, supplying ample substrates for microbial carbon mineralization [41]. Second, high Cd stress elevates CGR’s energy demand for Cd enrichment and detoxification, thus increasing root respiration rates [53]. The findings indicate that plant metabolic compensation demand increases with Cd stress intensity, aligning with previous evidence that heavy metal stress stimulates plant respiratory carbon emissions [54].

CGR regulates soil nitrogen cycling and thereby modulates N_2_O production [55]. In this experiment, its effect on N_2_O emissions showed a low-promotion, high-inhibition pattern with rising Cd concentrations, with AN dynamics as the core regulator. This pattern aligns with our hypothesis that CGR differentially affects GHG emissions. In Cd-5 soil, CGR significantly increased N_2_O emissions, likely via root exudates stimulating nitrifying and denitrifying bacteria—consistent with findings that invasive plants affect nitrogen transformation through rhizosphere microecology regulation [28,56]. However, at Cd-10 and Cd-30, enhanced AN uptake by CGR roots reduced soil NH_4_^+^-N and NO_3_^−^-N levels; below the microbial demand threshold, nitrification and denitrification were inhibited, cutting N_2_O emissions. Moreover, excessive Cd accumulation in CGR roots might suppress nitrogen-cycling microbial activity [57]. Overall, CGR’s regulatory effect on N_2_O emissions shifts from promotion to inhibition with increasing Cd concentrations, primarily due to root-exudate-mediated AN regulation and direct microbial inhibition by Cd stress. This highlights invasive plants’ environmental dependence regarding soil GHG emissions [52,58].

BC application regulates GHG emissions by modifying soil physicochemical properties, microbial community structure, and nutrient cycling [59]. In Cd-contaminated soils, BC remediation enhances short-term but strongly inhibits long-term CO_2_ emission rates; yet, cumulative CO_2_ emissions in BC treatments rise with increasing soil Cd concentrations, while remaining generally lower than those in the CGR remediation measure. This phenomenon links directly to Cd-driven shifts in carbon pool stability: BC is rich in aromatic carbon skeletons with low mineralization potential, which resist microbial degradation and protect soil organic carbon (SOC). Additionally, BC’s porous surface physically adsorbs DOC and particulate organic carbon (POC), reducing labile carbon availability for microorganisms and thus inhibiting SOC mineralization and CO_2_ emissions [60,61]. Notably, BC’s carbon sequestration effect is most pronounced under mild-to-moderate Cd contamination, while its CO_2_ emission inhibition weakens with rising Cd levels. This may be attributed to two factors: under high Cd stress, Cd-tolerant microorganisms secrete enzymes that partially degrade BC-adsorbed labile organic carbon, integrating it into mineralization; meanwhile, high Cd concentrations damage oxygen-containing functional groups (e.g., -OH, -COOH) on BC surfaces, lowering adsorption capacity [62]. In summary, the data show that BC’s efficacy as a carbon sink diminishes with higher Cd concentrations, consistent with the broader finding that elevated heavy metals disrupt carbon sequestration mechanisms via multiple pathways [63].

The impact of BC on N_2_O emissions was characterized by promotion at high Cd concentrations and no significant effect at low-to-medium levels, an outcome directly driven by Cd-regulated nitrogen species transformation. When Cd stress is moderate or mild, low ACd content enables BC to adsorb NH_4_^+^-N and NO_3_^−^-N via surface electrostatic attraction, which helps maintain soil nitrogen stability [64]. Additionally, BC raises soil pH and inhibits denitrifying bacteria activity; these two factors collectively lead to unchanged N_2_O emissions [65]. Conversely, under high Cd stress, ACd content rises sharply, and BC markedly accelerates N_2_O emissions. The promotion may be attributed to a dual pathway: (1) competitive displacement of adsorbed NH_4_^+^-N by Cd^2+^ on BC surfaces, which enhances nitrification substrate availability [66], and (2) inhibition of the N_2_O reductase enzyme under high Cd stress, blocking the reduction of N_2_O to N_2_ and causing its accumulation [67]. The impact of BC on N_2_O emissions was characterized by promotion at high Cd concentrations and no significant effect at low-to-medium levels, an outcome directly driven by Cd-regulated nitrogen species transformation. This study clarified the differential effects of *S. canadensis* and biochar on soil CO_2_ and N_2_O emissions across the Cd contamination gradient. However, as it did not include other GHGs, more comprehensive monitoring is needed in the future to systematically evaluate the climate impact of the remediation strategies.

## 5. Conclusions

The remediation performance of BC and CGR is highly dependent on Cd concentration. BC proves more effective under severe contamination (Cd-30), reducing ACd by 8.9% via increases in TC, DOC, and pH. Conversely, CGR shows a more targeted effect at lower concentrations, achieving substantial ACd reductions of 46.2% and 41.7% at Cd-5 and Cd-10, respectively, by modulating AN and DON. For GHG regulation, CGR increases cumulative CO_2_ emissions by 83.4% (Cd-10) and 53.8% (Cd-30), while BC inhibits N_2_O by 22.1% (Cd-5) and does not elevate CO_2_ across all levels, with better environmental potential. For balanced and effective remediation, CGR is advised for mild to moderate Cd contamination (≤10 mg kg^−1^), and BC for severe pollution (30 mg kg^−1^). This study not only offers a new direction for the resource utilization of invasive plants but also provides a quantitative basis and technical support to advance green remediation practices for Cd-contaminated soil. Based on the limitations of this study, future research should focus on the long-term field validation of these strategies, explore their synergistic effects in integrated applications, and monitor and assess multiple GHG emissions to inform optimal environmental outcomes.

## Figures and Tables

**Table 1:** Basic physical and chemical properties of test soil (means ± se). TC: total carbon; TN: total nitrogen; DOC: dissolve organic carbon; DON: dissolve nitrogen; NH_4_^+^-N: ammonium nitrogen; NO_3_^−^-N: nitrate nitrogen; ACd: available cadmium, the same below.

Test Materials	pH	TC (g kg^−1^)	TN (g kg^−1^)	DOC (mg kg^−1^)	DON (mg kg^−1^)	NH_4_^+^-N (mg kg^−1^)	NO_3_^−^-N (mg kg^−1^)	ACd (mg kg^−1^)
Soil	7.93 ± 0.09	8.22 ± 0.15	1.29 ± 0.13	4.18 ± 0.93	0.56 ± 0.05	5.04 ± 0.29	7.08 ± 0.58	0.12 ± 0.03

**Table 2:** The effects of remediation measures and pollution levels on soil properties in ANOVAs. Remediation measures: control (CK), biochar (BC), and *S. canadensis* planting (CGR); Cd: 0 (Cd-0), 5 (Cd-5), 10 (Cd-10), and 30 (Cd-30) mg kg^−1^, respectively. *F* with *p* values indicated by asterisks. ***, *p* < 0.001.

Factors	Remediation Measures	Cd	Remediation Measures × Cd
df	2	3	6
pH	54.693 ***	21.508 ***	0.934
TC (g kg^−1^)	979.544 ***	14.118 ***	15.111 ***
TN (g kg^−1^)	234.468 ***	47.457 ***	8.977 ***
DOC (mg kg^−1^)	0.642	6.054 ***	3.158
DON (mg kg^−1^)	35.95 ***	6.078 ***	2.007
NH_4_^+^-N (mg kg^−1^)	0.048	10.491 ***	0.5
NO_3_^−^-N (mg kg^−1^)	21.168 ***	1.623	0.472
AN (mg kg^−1^)	19.571 ***	0.723	0.894
ACd (mg kg^−1^)	12.211 ***	1343.782 ***	1.812
TC/TN	1138.322 ***	29.38 ***	17.036 ***
DOC/DON	88.529 ***	4.326	0.237

**Table 3:** The effects of remediation measures, cadmium (Cd) pollution level, and their interaction on greenhouse gas emissions in ANOVAs. *, *p* < 0.05; ***, *p* < 0.001.

Factors	Remediation Measures	Cd	Remediation Measures × Cd
*df*	2	3	6
N_2_O emission rate (μg m^−2^ h^−1^)	0.887	0.688	0.658
CO_2_ emission rate (mg m^−2^ h^−1^)	25.731 ***	24.053 ***	1.901
Cumulative N_2_O emission (mg m^−2^ h^−1^)	8.52 ***	1.79 *	8.904 ***
Cumulative CO_2_ emission (g m^−2^ h^−1^)	116.42 ***	50.058 ***	7.005 ***

## Data Availability

Data are contained within the article.
